# Lake sedimentary biogenic silica from diatoms constitutes a significant global sink for aluminium

**DOI:** 10.1038/s41467-019-12828-9

**Published:** 2019-10-23

**Authors:** Dong Liu, Peng Yuan, Qian Tian, Hongchang Liu, Liangliang Deng, Yaran Song, Junming Zhou, Dusan Losic, Jieyu Zhou, Hongzhe Song, Haozhe Guo, Wenxiao Fan

**Affiliations:** 1 CAS Key Laboratory of Mineralogy and Metallogeny/Guangdong Provincial Key Laboratory of Mineral Physics and Materials, Guangzhou Institute of Geochemistry, Institutions of Earth Science, Chinese Academy of Sciences, 510640 Guangzhou, China; 2 University of Chinese Academy of Sciences, 100049 Beijing, China; 3 School of Chemical Engineering, University of Adelaide, Adelaide, SA 5005 Australia

**Keywords:** Element cycles, Element cycles, Environmental chemistry, Limnology

## Abstract

Diatoms play an important role in marine biogeochemical cycle of aluminum (Al), as dissolved Al is taken up by diatoms to build their siliceous frustules and is involved in the sedimentation of diatomaceous biogenic silica (BSi). The Al incorporation in BSi facilitates decreasing the dissolution of marine BSi and thus substantially influences the biochemical processes driven by diatoms, such as CO_2_ sequestration. However, the role of lake BSi in the terrestrial biochemical Al cycle has not been explored, though lakes represent the second-largest sink for BSi. By identifying the previously unexplored high Al/Si atomic ratios (up to 0.052) in lake BSi, here we show lake BSi is a large terrestrial Al pool due to its high Al content, and lake sedimentary BSi constitutes a significant global sink for Al, which is on the same magnitude as the Al sink in global oceans.

## Abstract (web summary)

Diatoms drive biogeochemical cycling of aluminum by incorporating this element into their shells, but this process has not been quantified in freshwater systems. Here the authors quantify diatom-mediated aluminum fluxes in lakes and determine that they rival the aluminum sink in the global ocean.

## Introduction

Aluminium (Al) is the third most abundant element in the Earth’s crust. Its biogeochemical cycling on land, in water, and in the atmosphere is a central topic of discussion in many geochemical, environmental science, and biological studies^1^ due to the active role of Al in geochemical processes (e.g., weathering and reverse weathering) as well as the ecological and environmental impacts of Al (e.g., potential ecotoxicity to organisms)^2,3^. The marine biogeochemical cycling of Al is strongly associated with the cycling of silicon (Si) through marine diatoms^4–10^. Diatoms account for ~40% of marine primary production^11,12^, and the gross production of biogenic silica in the ocean reaches ~2.40 × 10^14^ mol year^−1^ (refs. ^13,14^). Diatoms control the biogenic cycling of Si throughout the world’s oceans because they use Si to build siliceous frustules^11,15^. The inorganic part of diatom is diatomaceous biogenic silica (denoted BSi hereafter)^16^. The coupled Al and Si biogeochemical cycle occurs primarily during BSi biosynthesis, in which Al is incorporated into the structure of BSi with Al/Si atomic ratios of up to ~0.008 (ref. ^8^). The marine BSi subsequently settles, and ~3% of the total marine BSi is eventually preserved in the seafloor (with a burial rate of ~6.30 × 10^12^ mol year^−1^)^14^. This process exports organic carbon to the deep sea through the marine biological pump and is therefore important for CO_2_ sequestration^12^. Furthermore, the process also delivers a large amount of dissolved Al from marine waters to the seafloor^17,18^. The magnitude of Al deposition associated with marine BSi is nearly equivalent to that of the riverine input of dissolved Al into the oceans^17^, and constitutes an important part of the marine geochemical Al cycle^18^.

While marine Al cycle in relation to marine BSi has been well studied^19^, surprisingly, no reports have considered diatoms-driven Si and Al co-cycles in lakes, even though lakes represent the second-largest sink of BSi after the oceans and the rate of BSi burial in global lakes reaches ~1.30 × 10^12^ mol year^−1^ (ref. ^20^). This rate is on the same order of magnitude as that of marine BSi burial, although the area of lakes (~4.20 × 10^6^ km^2^) is significantly smaller than that of oceans (~2.40 × 10^9^ km^2^) where BSi-containing sediments occur^14,20^. Moreover, lakes have significantly higher concentrations of dissolved Al than oceans^1,3,21^. For example, the average Al concentration of over 400 freshwater lakes (with pH ≥ 5.0) in USA is higher than 1.0 μM^3^. These concentrations are several orders of magnitude greater than those measured in the oceans^19,22–26^; e.g., the dissolved Al concentrations are ~0.3‒5.0 nM in the surface waters of the Pacific Ocean^24^ and rarely exceed 20.0 nM in the surface water of open ocean^19,22,25^. Given the high Al concentration in lakes and the large rate of burial of lake BSi, we hypothesize that the scale of Al uptake by diatoms and the Al burial due to BSi could differ significantly between lakes and oceans. Quantifying the uptake of dissolved Al by lake BSi is therefore crucial to address this question. In particular, identifying the level of Al incorporation in the structures of lake BSi, which has not been explored previously, is key for estimating the magnitude of Al burial in relation to BSi in global lakes, as well as for assessing the role of lake BSi in the terrestrial Al cycle.

Here we show that lake BSi has high ratios of Al incorporation in its structure by identifying the level and state of Al incorporation, using a combination of cross-section and surface analysis techniques with high sensitivity, such as focused ion beam (FIB) milling combined with electron microscopy equipped with energy-dispersive X-ray spectroscopy (EDS) and time-of-flight secondary ion mass spectrometry (TOF-SIMS). Our results demonstrate that the proportion of structural Al in lake BSi is high (with Al/Si atomic ratio being up to 0.052), which is significantly higher than the maximum Al/Si atomic ratio (~0.008 (ref. ^8^)) of marine BSi. Based on these results, we estimate that the burial rate of Al through Al incorporation in BSi in natural lakes globally is ~1.72 × 10^9^ kg year^−1^, which is on the same order of magnitude as that of the marine Al burial rate (~1.36 × 10^9^ kg year^−1^). Therefore, lake BSi is a large terrestrial Al pool due to the high Al content in lake BSi, and lake’s sedimentary BSi constitutes a significant global sink for Al. This large Al retention due to lake BSi needs to be acknowledged in the modelling of the global biogeochemical Al cycle that is currently not considered.

## Results

### Identification of Al distribution and coordination in BSi

**Fig. 1:**
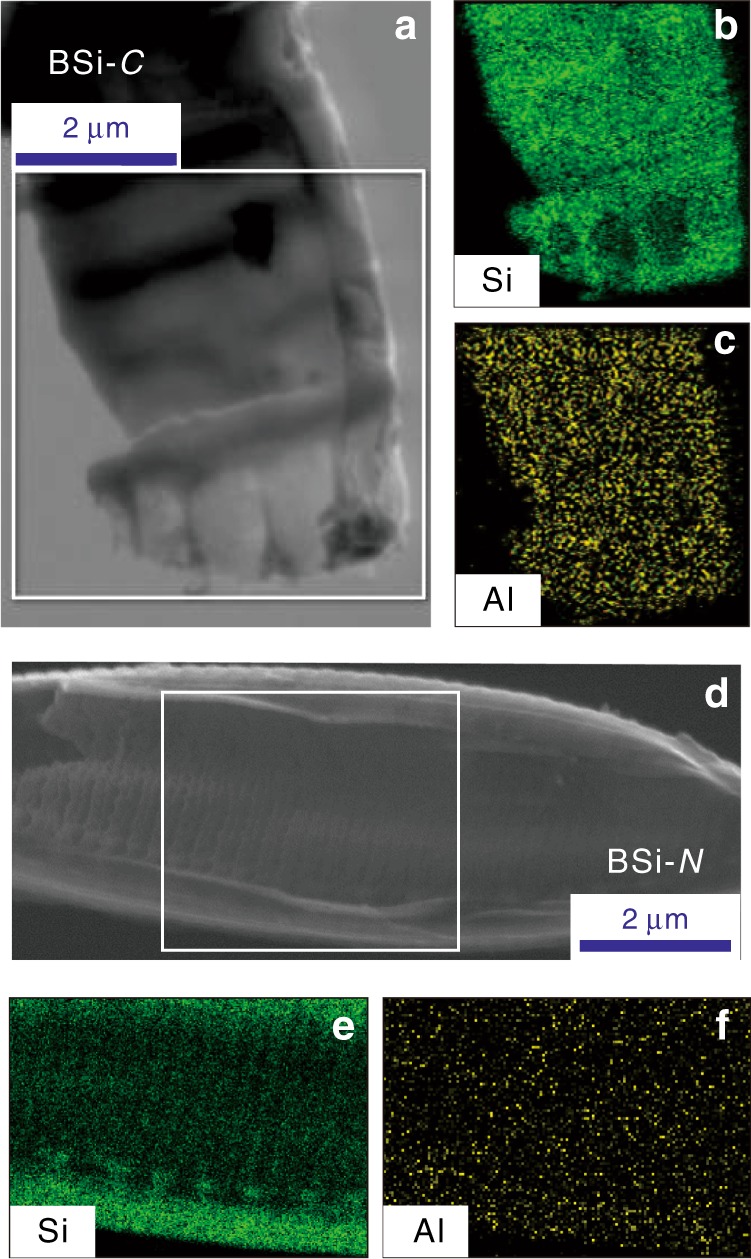
Morphology and composition of the interior of diatom frustules. **a** Field-emission scanning electron microscope (FESEM) image of the typical morphology of a frustule in BSi-*C*, pretreated by FIB milling. **b** Energy-dispersive X-ray spectroscopy (EDS) mapping of Si distribution (green shading) in the frustule of BSi-*C*. **c** EDS mapping of Al distribution (yellow shading) in the frustule of BSi-*C*. **d** FESEM image of a frustule in BSi-*N*, pretreated by FIB milling. **e** EDS mapping of Si distribution (green shading) in the frustule of BSi-*N*. **f** EDS mapping of Al distribution (yellow shading) in the frustule of BSi-*N*

Two model species of lake diatoms, *Cyclotella meneghiniana* (Bacillariophyceae: *Centricae*) (denoted BSi-*C*) and *Nitzschia palea* (*Pennatae*) (BSi-*N*), which are among the most widely distributed freshwater diatoms in the world^27^, were used for studying the uptake of Al by lake BSi. The Si and Al distributions in the BSi structures were examined using FIB milling (see Supplementary Fig. 1 for the details of the FIB pretreatment of BSi-*C* as an example) combined with EDS mapping analysis (Fig. 1). This FIB milling process allows detection of the inner structure of the BSi, thus avoiding possible disturbances from non-structural Al on the external surface of the BSi. The FIB–EDS mapping images of the BSi-*C* (Fig. 1c) and BSi-*N* (Fig. 1f) show that Al is homogeneously distributed in the siliceous structure of lake BSi.

**Fig. 2:**
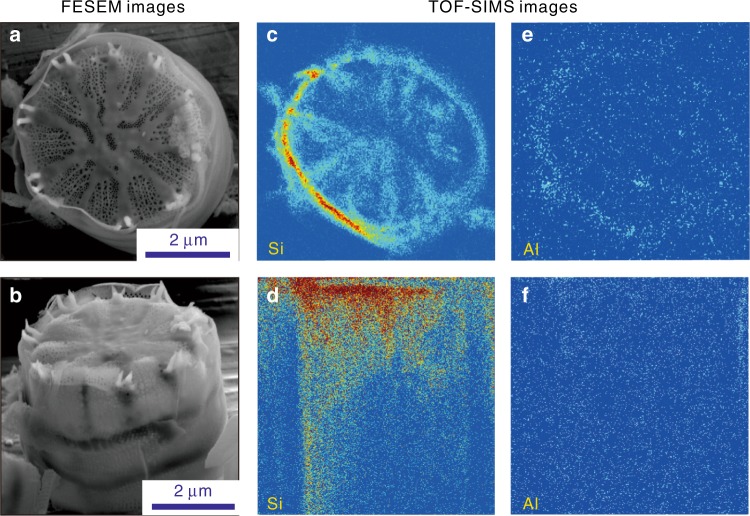
Morphology and composition of a frustule of BSi-*C*. **a** Top view and **b** isometric view (tilted 55° from the top view) of the frustule of BSi-*C* in the field-emission scanning electron microscope (FESEM) images. **c** Top view and **d** side view of the three-dimensional (3D) Si distributions obtained by time-of-flight secondary ion mass spectrometry (TOF-SIMS). **e** Top view and **f** side view of the 3D Al distributions obtained by TOF-SIMS. The signal fading from left to right in the 3D images in (**c**) and (**d**) was caused by the edge effect; i.e., the primary ion beam came from the left side and resulted in unhomogenized signals due to the round shape of the frustule^41^

To evaluate the 3D structural Al distributions in BSi, TOF-SIMS analysis producing 3D reconstructions of architectural elements was used^28^. The TOF-SIMS analysis result of a frustule of BSi-*C* exemplifies the 3D elemental distribution. A typical disk-like *C. meneghiniana* frustule with a diameter of ~4.0‒5.0 μm (determined by field-emission scanning electron microscope (FESEM) imaging; Fig. 2a) and a thickness of ~2.5 μm (Fig. 2b) was subjected to the 3D elemental characterization by TOF-SIMS. Figures 2c–f show the Si and Al distributions in the top view and side view of the frustule of BSi-*C*. Al is clearly homogeneously distributed throughout the structure of the BSi, which is further indicated by the visible 3D Si and Al distributions from all angles (see Supplementary Movie for a short video of the reconstructed elemental distributions). This result demonstrates the incorporation of Al into the structure of the BSi of lake diatoms.

**Fig. 3:**
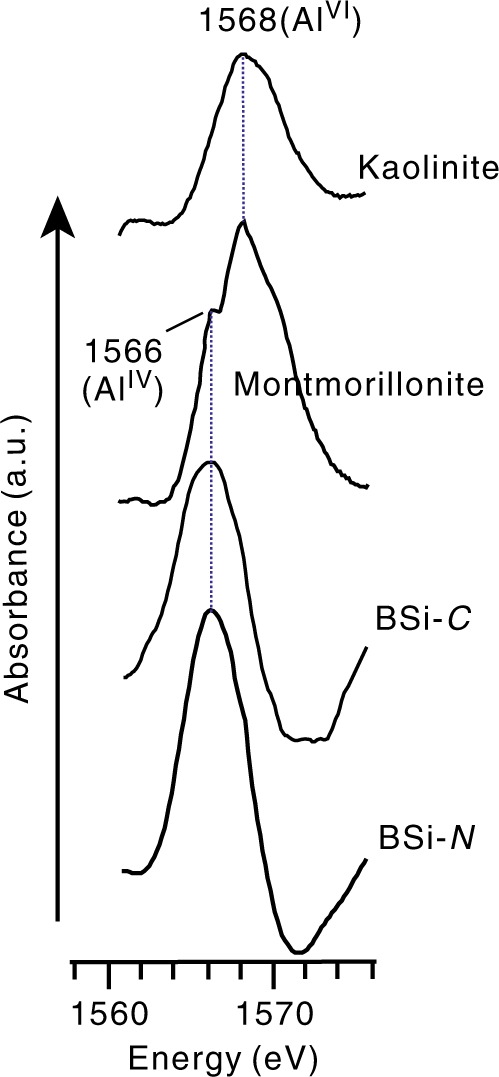
Al coordination states shown by X-ray absorption spectroscopy. Al K-edge X-ray absorption near-edge structure (XANES) spectra of BSi-*C*, BSi-*N*, and reference minerals (montmorillonite and kaolinite) were obtained to detect the Al coordination states. Montmorillonite and kaolinite are selected as reference compounds for minerals containing a mix of four- and sixfold coordinated Al (montmorillonite) and sixfold coordinated Al only (kaolinite); neither of these minerals is of biogenic origin. The XANES spectrum of kaolinite shows a single energy signal at 1568 eV, which is contributed by octahedral Al^29^. Montmorillonite has a strong signal at 1568 eV and a weak signal at 1566 eV, which corresponds to octahedral and tetrahedral Al, respectively^30^. Al^IV^ and Al^VI^ represent four- and sixfold coordinated Al, respectively. Source data are provided as a Source Data file

Al K-edge X-ray absorption near-edge structure (XANES) spectroscopic analysis was used to reveal the coordination states of the structural Al in the lake BSi. The XANES spectra (Fig. 3) indicate that the Al in the lake BSi exists mainly in tetrahedral coordination, regardless of the diatom species, as evidenced by the 1566-eV signal of tetrahedral Al^29,30^. Isomorphous Si substitution by Al therefore occurs in natural lake BSi because the Si in BSi is tetrahedrally coordinated, verifying the incorporation of Al into lake BSi through Al–Si substitution. This incorporation mechanism of Al into BSi implies that the state of Al in BSi is stable because isomorphous substitution is significantly stronger than non-structural incorporation (such as by adsorption), as is well known.

### Quantification of structural Al in BSi and in diatoms

**Fig. 4:**
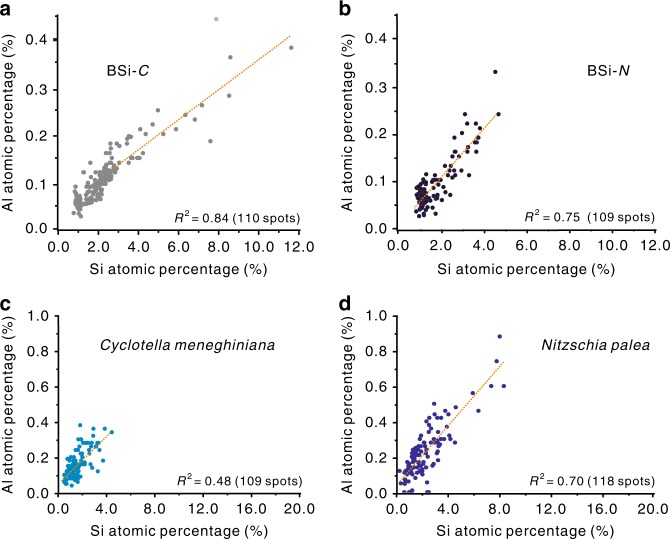
Al and Si atomic percentages in frustules and entire diatoms. **a** The data of Al and Si atomic percentages for BSi-*C*; **b** the data for BSi-*N*; **c** the data for the entire *Cyclotella meneghiniana* diatoms; and **d** the data for the entire *Nitzschia palea* diatoms. Energy-dispersive X-ray spectroscopy (EDS) spot analysis was used for the determination, and the data of more than 100 spots (specified in the brackets of **a**‒**d**) from over 35 entire diatoms or their BSi were collected. *R*^2^ is the correlation coefficient between the atomic percentages of Al and Si (the linear fitting formulas are given in Supplementary Table 1). Source data are provided as a Source Data file

FESEM with EDS spot analysis was used to determine the atomic percentages of Al and Si in lake BSi. Over 100 spots from different frustules of lake BSi for each diatom species were collected for the spot analysis. The results reveal high Al incorporation capacities for all of the lake BSi samples. Based on the atomic percentages of Al and Si in the BSi (Fig. 4a, b), the average lake Al/Si atomic ratios (Al/Si_BSi_) are 0.049 ± 0.003 and 0.052 ± 0.003 for BSi-*C* and BSi-*N*, respectively (Supplementary Fig. 2)—at least six times greater than the maximum Al/Si_BSi_ ratio (~0.008)^8^ of natural marine BSi. The Al/Si ratios of the entire diatoms (i.e., the intact diatoms, in which the organics were not removed) are 0.105 ± 0.008 (*C. meneghiniana*) and 0.115 ± 0.012 (*N. palea*) (Supplementary Fig. 2). Positive correlations (*R*^2^ ≥ 0.75) are observed between the Si and Al contents of BSi for the lake BSi samples (Fig. 4a, b), indicating the incorporation of Al into the BSi of natural lake diatoms. A similar positive correlation as an indicator of Fe incorporation into marine BSi has been clarified previously^31^.

**Fig. 5:**
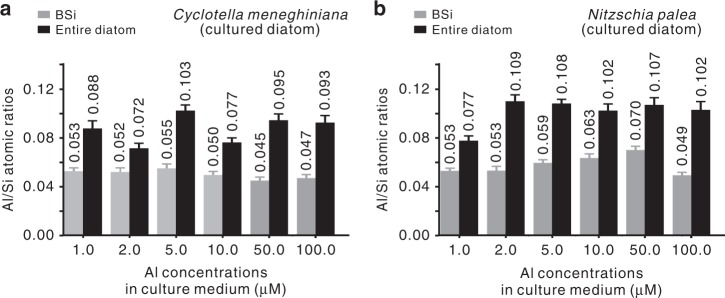
Al/Si atomic ratios of cultured diatoms and their frustules. **a** The average Al/Si atomic ratios of the entire *Cyclotella meneghiniana* diatoms (black columns) cultured in media with various Al concentrations and of their biogenic silica (BSi) (grey columns). **b** The data of the entire *Nitzschia palea* diatoms (black columns) and of their BSi (grey columns). The energy-dispersive X-ray spectroscopy (EDS) spot analysis for the determination of Al/Si atomic ratios is based on over 100 spots for each sample. Error bounds were obtained on the basis of 95% confidence interval for the analysis on Al/Si atomic ratios. Source data are provided as a Source Data file

We investigated the influence of the Al concentration in the culture medium on the Al/Si_BSi_ values of cultured diatoms by EDS spot analysis. The cultured diatom species are the same as the abovementioned natural lake diatoms. Six Al concentrations (1.0, 2.0, 5.0, 10.0, 50.0, and 100.0 μM) were applied, with Si concentrations remaining constant. The obtained Al/Si_BSi_ values range between 0.045 and 0.055 for cultured *C. meneghiniana* and between 0.049 and 0.070 for cultured *N. palea* (grey columns in Fig. 5a, b). In line with the observations for natural lake BSi, the Al/Si_BSi_ values of cultured lake BSi are also significantly higher than those of the BSi of cultured marine diatoms^8,32–34^, which showed a maximum Al/Si_BSi_ value of only ~0.007 (for the marine *Lauderia annulata* diatoms with an Al concentration of 400 nM in the culture medium)^8^. In addition, similar to those of natural lake diatoms (Supplementary Fig. 2), the Al/Si_BSi_ values of cultured diatoms are lower than the Al/Si values of the entire diatoms (Fig. 5). This pattern occurs because Al exists not only in BSi but also in the organic components of diatoms^4^. This point is supported by the mass spectrometry (MS) data from TOF-SIMS (see Supplementary Fig. 3) analysis of the cultured *C. meneghiniana*, from which the Al species associated with the organic components of the Al-bearing lake diatoms were determined. Notably, the specific mechanisms, explaining why diatoms take up Al into their frustules and how Al is transported or distributed between the inorganic and organic components of diatoms are unknown and have never been explored^35^.

As the Al concentrations in the culture medium increased from 1.0 to 100.0 μM, no obvious variation in the obtained Al/Si_BSi_ values for the two types of cultured BSi is observed. This result suggests that the lowest Al concentration (1.0 μM) may be sufficient to permit the incorporation of Al into the BSi of the freshwater diatoms and that high environmental Al concentrations do not further increase the incorporation of Al into lake BSi. Furthermore, two Al concentrations (1.0 and 2.0 μM) in the culture medium were selected for comparison. These concentrations are similar to the Al concentrations in the natural lakes from which *C. meneghiniana* and *N. palea* were collected (1.1 and 2.1 μM, respectively, see Supplementary Table 2). The average Al/Si_BSi_ values of cultured and lake BSi are similar—0.053 versus 0.049 (*C. meneghiniana*), respectively; and 0.053 versus 0.052 (*N. palea*), respectively. Therefore, the cultured lake BSi and natural lake BSi exhibit a similar feature of a high proportion of structural Al, which is not noticeably affected by the environmental Al concentration or the species of diatom. In addition, FIB–EDS analysis was used to detect Al incorporation into the internal structure of BSi in the lake sediments. The results show the homogenous Al distribution in the bulk of BSi of lake sediments (Supplementary Fig. 4), and the average Al/Si_BSi_ values of two frustules of *C. meneghiniana* and *N. palea* are ~0.047 and ~0.051, respectively.

## Discussion

The small variations in Al/Si_BSi_ values among the BSi of the lake diatoms cultured with different Al concentrations suggest that the Al/Si_BSi_ values for lake BSi are relatively constant at common Al concentrations (at a level of several micromolars^1^) in non-acidified lakes. Furthermore, the Al/Si_BSi_ values of the BSi from living lake diatoms are similar to those from the lake sediments. This feature is a result of the nature of the isomorphous Al–Si substitution in BSi, which guarantees a homogenous Al distribution and leads to relatively constant Al/Si_BSi_ value, either in local or in bulk of BSi. Therefore, the remarkably high degree of Al incorporation (Al/Si_BSi_ values up to 0.052) in natural lake BSi substantiates its role as a significant Al pool in the terrestrial Al cycle. We adopt the average Al/Si_BSi_ value (0.049) of BSi-*C* as a representative value to estimate the Al retention due to BSi in lakes. Notably, such an estimate is conservative because the adopted Al/Si_BSi_ value is situated in the low region of all the Al/Si_BSi_ values determined. Additionally, the Al sink due to lake BSi in a certain lake depends on the actual burial rate of BSi, which is influenced by the chemical and ecological conditions of the lake. For lakes globally, the total burial of BSi is ~1.30 × 10^12^ mol year^−1^ (ref. ^20^). Consequently, the Al retention due to the burial of BSi in global lakes is estimated to be ~1.72 × 10^9^ kg year^−1^. By comparison, the burial of Al in BSi in the global oceans is ~1.36 × 10^9^ kg year^−1^, as estimated from the rate of burial of Si in the global oceans of ~6.30 × 10^12^ mol year^−1^ (ref. ^14^) and the highest Al/Si_BSi_ value (~0.008)^8^ in marine BSi. In other words, the magnitude of retention of Al due to the burial of BSi in terrestrial lakes globally is comparable to (or even possibly larger than) that in the global oceans.

From the perspective of the biogeochemical Al cycle, the abovementioned Al burial due to lake BSi accounts for ~9.1% of the total export of terrestrial Al in biomass to continental sediments (~1.90 × 10^10^ kg year^−1^)^36^. Moreover, the magnitude of this Al sink is comparable to the magnitude ((0.4‒2.9) × 10^9^ kg year^−1^ (ref. ^18^)) of the input of dissolved Al into oceans by rivers globally. Based on the burial rate of Al due to BSi (~1.72 × 10^9^ kg year^−1^) in global lakes and on an estimate that ~5‒20% of total lake BSi is eventually buried in sediments^37^, the total Al uptake by the BSi of global lakes is on the order of (~0.86‒3.44) × 10^10^ kg year^−1^. Therefore, the Al occurrence in lake BSi, either in the waters or in the sediment, constitutes an important part of the global Al cycle.

The large Al pool due to Al incorporation in lake BSi, especially the Al sink through the burial of lake BSi, has not been considered in the scientific literature, but may have substantial effects on the global Al cycle. The scale of the terrestrial dissolved Al exported to oceans could be affected by the magnitude of Al burial in lakes, thereby mediating related marine geochemical processes, such as reverse weathering in which Al ions are active reactants^2^. For modelling and interpretations of the global Al cycle, the Al uptake by lake BSi and its potential consequences must be taken into account.

Al incorporation into amorphous silica has long been known to be able to reduce silica solubility^5,7–9,38^. The decrease in solubility of the BSi of cultured marine diatoms with Al incorporation was also confirmed^8^. The mechanism of the abovementioned dissolution–inhibition effect has been ascribed to the production of negative charges by Al incorporation in silica, which repels OH^−^ and thereby retards the dissolution of silica^7–9,38^. The Al/Si_BSi_ values of the BSi of marine diatoms normally remain at a level of 10^−4^‒10^−3^ in marine waters with generally low Al concentrations^33^. The low extent of Al incorporation in marine BSi is thought to be unable to obviously affect the dissolution kinetics or the solubility of marine BSi^6,9^. In contrast, the noticeable Al incorporation in lake BSi substantially decreases its solubility. In a 10-day dissolution test aiming at examining the dissolution–inhibition effect of Al-bearing lake BSi, the Al-scarce BSi showed high dissolution extent, 90.2% (wt%) for the BSi from the cultured *C. meneghiniana* and 79.6% for that from the cultured *N. palea* (details in Supplementary Fig. 5). However, the 10-day dissolution extents of the Al-bearing BSi with different Al/Si_BSi_ values decreased to 49.6‒67.7% and 55.4‒66.6% (Supplementary Fig. 5), for the cultured *C. meneghiniana* and *N. palea*, respectively.

The consequences of such a distinct dissolution–inhibition effect of lake BSi deserve to be considered in the preservation of lake BSi. The annual burial rate of lake BSi normalized to the total area of global lakes is 18.2 g m^−2^ year^−1^ (ref. ^20^). This value is ~11.5 times greater than that of marine BSi (1.58 g m^−2^ year^−1^) normalized to the area (~2.40 × 10^9^ km^2^) of BSi-containing oceanic regions^14^. Given the key role of diatoms in the closely coupled Si and C cycles through the biological pump^12^, this result suggests that the biological pump driven by diatoms in lakes is more efficient than its counterpart in oceans, making the former biological pump more conducive to CO_2_ sequestration. The pronounced dissolution–inhibition effect likely contributes to the high efficiency of sedimentation and preservation of lake BSi, although quantifying this contribution is difficult at the present stage due to the lack of data of other variables, such as the scale of biological scavenging^6^. The role of lake BSi for carbon sequestration over geological history or during the present, and the potential impacts by the preservation of lake BSi on mediating global climate change, warrants further study.

## Methods

### Lake diatoms

Two species of diatom, *C. meneghiniana* (Bacillariophyceae: *Centricae*) and *N. palea* (*Pennatae*), were collected from Wuliangsuhai Lake and Taihu Lake in China, respectively. These samples are referred to as natural lake diatoms. Site descriptions and sampling procedures are provided in the Supplementary Materials (Supplementary Figs. 6 and 7 and the associated text). A plankton net with a 0.22-μm mesh was used to collect the living diatoms of *C. meneghiniana*, and a Teflon collector was used for *N. palea*. The living diatoms were picked from the collected specimens using an Olympus Microscope (CX31) at 1000× magnification. The identification of the species of the living diatoms was achieved by determining the rbcL gene sequences of the diatoms^39^ (Supplementary Fig. 8). Before the analysis such as determination of Al/Si atomic ratios through EDS analysis, diatom samples were cleaned in 0.1 N citric acid for 72 h and rinsed three times with deionized water, and the products were subjected to centrifugation and subsequent freeze-drying.

### Cultivation of diatoms

Cultured diatoms of the same two species as the abovementioned living diatoms were provided by the Freshwater Algae Culture Collection at the Institute of Hydrobiology (FACHB), Chinese Academy of Sciences. After successive culturing and harvesting over many generations in an Al-free medium, these diatoms contain very little Al (Al/Si atomic ratios <0.001). These Al-scarce diatoms were used as control samples for comparison. The diatoms were grown in the culture medium (details of the components are given in Supplementary Table 3) at a temperature of 25 °C under a 12/12 light/dark cycle at an intensity of 71 μmol photons m^−2^ s^−1^ for 480 h (one diatom life cycle). The pH of the diatom culture medium was adjusted to 8.0 using a solution of NaOH. This pH is close to the pH of the abovementioned lakes where the natural lake diatoms were collected. The pH of the culture medium during culture process is in the range of 8.0–8.4. Al-bearing diatoms were obtained by adding AlCl_3_ to the diatom medium to reach designated Al concentrations of 1.0, 2.0, 5.0, 10.0, 50.0, and 100.0 μM. The diatoms cultured in the abovementioned weakly alkaline condition of this work have a high level of Al tolerance, unlike the pronounced ecotoxicity of Al to organisms shown at the acidic or circumneutral conditions^40^. The growth inhibition of the diatom cells in the presence of Al was found to be minor, even for the high Al concentrations of 50.0 and 100.0 μM (Supplementary Fig. 9). The cultured diatoms were treated according to the same procedures described above before further analysis, such as microscopic characterization.

### Extraction of BSi

Two chemical treatments were used to obtain the BSi samples from the natural lake diatoms and the cultured diatoms, including immersion in a 30% hydrogen peroxide solution for 72 h to remove the organic matrix of the diatoms, and then washing in 0.1 N acetic acid for 72 h to remove non-structural Al^4^. These steps were repeated until complete removal of the organic components was achieved. To confirm the removal of organics, carbon elemental analysis on the BSi was performed using a Vario EL III elemental analyzer, in which samples were oxidized in a combustion tube in the presence of oxygen at high temperature (≥1150 °C). Samples of ~2.00 mg were placed in tin boats with a size of 6 × 12 mm. The carbon contents of the BSi extracted from the diatoms were all below the detection limit (<40 ppm).

### Determination of Al/Si atomic ratios

The Al/Si atomic ratios in the natural lake diatoms and the cultured diatoms and their BSi were determined by an EDS method using a Hitachi SU8010 FESEM attached to an AMETEK energy dispersive X-ray spectrometer. The samples for SEM characterization were pre-treated by carbon coating. Over 100 spots from more than 35 entire diatoms and their BSi of each diatom species were detected using FESEM–EDS spot analysis with a voltage of 15 kV and a current of 20 μA. For the cultured diatoms and their BSi, 103 spots of EDS spot analysis for each sample were collected.

### FIB–EDS mapping analysis

FIB–EDS mapping analyses of the natural lake BSi were carried out to reveal the Al and Si distributions in the BSi samples. This process was achieved using a FIB–SEM (GAIA3, Tescan) equipped with a Triglav FESEM column, a Cobra FIB column, a multichannel gas injection system, and an Oxford Instrument X-Max 80 Aztec energy-dispersive X-ray spectrometer. A single frustule of BSi selected for characterization was picked using a nanomanipulator (Oxford OmniProbe 400), and the sample surface was milled using a focused gallium ion (Ga^+^) beam with an accelerating voltage of 30 kV, a current of 1.0 nA, and a total milling time of 1 min.

### TOF-SIMS analysis

A frustule of *C. meneghiniana* BSi (BSi-*C*) was analysed by TOF-SIMS to identify the 3D elemental distribution of Al and Si in the BSi. The SIMS instrument is equipped with a reflection-type TOF analyser (GAIA TOF-SIMS, Tescan); a liquid metal ion gun with 30 keV of Ga^+^, an ion beam current of 25 pA, and an angle of incidence to the normal of 55° for bombarding the surface; and an electron beam for imaging. Each layer of BSi-*C* is produced with an ion beam for surface analysis, and a sputtering ion beam is used to ablate the surface layer^28^.

### Al K-edge XANES analysis

Al K-edge XANES analysis was performed on the BL08U beamline of the Shanghai Synchrotron Radiation Facility (SSRF; Shanghai, China). A dispersion was prepared by mixing BSi with ultrapure water and subsequently shaking the mixture to disperse the samples. The dispersion was dropped onto tinfoil and smeared evenly. After the dispersion was air-dried, the tinfoil coated with BSi was positioned at a 90° angle to the incident X-ray beam. Al K-edge XANES spectra were recorded in total electron yield mode in a vacuum chamber (<10^−5^ torr, 25 °C) from 1558 eV to 1576 eV across the Al K-edge with a step of 0.1 eV and a dwell time of 2 s, and the measurement was repeated three times at each energy level. The XANES spectra were then normalized with the IFEFFIT program.

### Dissolution experiment of Al-bearing BSi

To determine the effect of Al incorporation on the solubility of BSi, the BSi samples extracted from the cultured *C. meneghiniana* and *N. palea* were used. A series of BSi samples with average Al/Si atomic ratios ranging from <0.001 (Al-scarce BSi) to 0.045–0.070 (Al-bearing BSi) were used for the dissolution experiment; 0.50 mg of BSi sample was dissolved in 100 mL of deionized water in a Teflon beaker at 25 °C. After 10 days of dissolution, the resulting solution was centrifuged and 1.0 mL of solution from the supernatant was collected for the determination of Si concentration, which was performed using a Thermo Scientific iCAP 7000 inductively coupled plasma atomic emission spectrometer (ICP-AES). Before ICP-AES analysis, 1.0 mL of 2.0% HNO_3_ was added into the solution and the resultant extract solution was left overnight. The parameters of ICP-AES were as follows: a power of 1150 W, a plasma flow rate of 12 L min^−1^, a coating gas flow rate of 0.5 L min^−1^, and a nebulisator pressure of 200 kPa. Digestions of the blank sample were simultaneously carried out for calibration. The 10-day dissolution extent of BSi was calculated using the following equation:

$$D(\% ) = \frac{{\frac{{\frac{{M \times 2.0 \times 10^{ - 3}}}{{28.1 \times 10^{ - 3}}} \times 60.1 \times 10^{ - 3}}}{{1.0 \times 10^{ - 3}}} \times 100 \times 10^{ - 3}}}{{0.5}} \times 100$$

where *D* (%) is the 10-day dissolution extent of BSi, *M* (mg L^−1^) is the concentration of Si detected by ICP-AES, and the atomic weights of Si and SiO_2_ are 28.1 and 60.1 g mol^−1^, respectively.

### Reporting Summary

Further information on research design is available in the Nature Research Reporting Summary linked to this article.

## Supplementary information

Supplementary Information

Supplementary Movie: Three-dimensional Si and Al distributions of a frustule in BSi-C

Reporting Summary

Source Data

## Data Availability

The authors declare that the main data supporting the findings of this study are available within the article and its Supplementary Information files. Extra data are available from the corresponding author upon request. The source data underlying Figs. 3, 4, and 5 and Supplementary Figs. 2, 3, 5, and 9 are provided as a Source Data file.

## Supplementary information

**Supplementary information** is avaliable for this paper at 10.1038/s41467-019-12828-9.
