# Supplementary material for: Lake sedimentary biogenic silica from diatoms constitutes a significant global sink for aluminium
Source: Nat Commun. 2019 Oct 23;10:4829. doi: 10.1038/s41467-019-12828-9 (PMC6811591; doi:10.1038/s41467-019-12828-9)
Supplement: Supplementary file 1 — Supplementary Information [file 41467_2019_12828_MOESM1_ESM.pdf]

Supplementary Information for Lake sedimentary biogenic silica from diatoms constitutes a significant global sink for aluminium by Liu *et al*.

Supplementary Figures

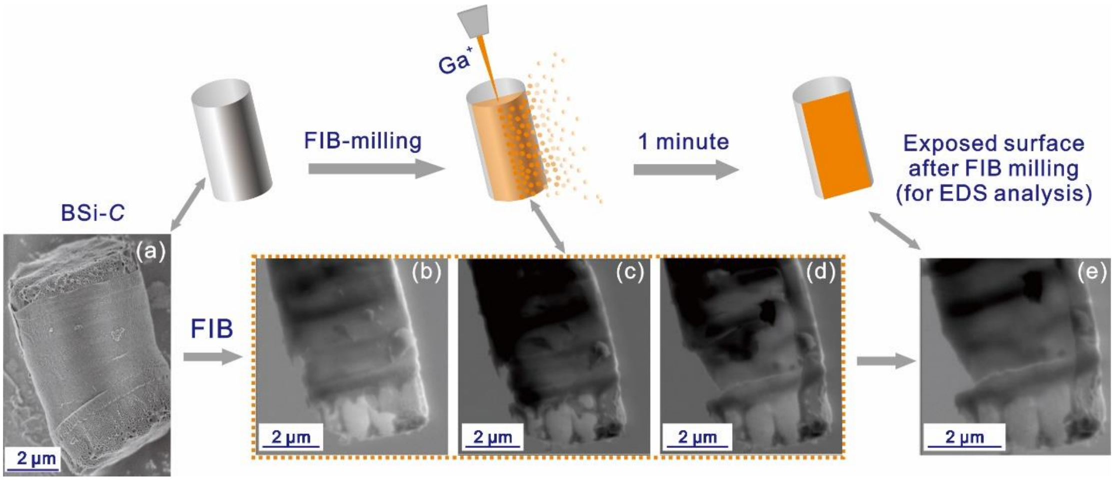

Supplementary Figure 1. FESEM images of (a) a frustule of BSi-*C* (lake BSi of *Cyclotella meneghiniana*) and (b-e) its serial focused ion beam (FIB) milling products during one-minute ion sputtering experiments. The images show that the external surface layers of BSi-*C* were gradually ablated by ion sputtering, and the internal layers were exposed for further energy-dispersive X-ray spectroscopy (EDS) mapping of the elements of Al and Si.

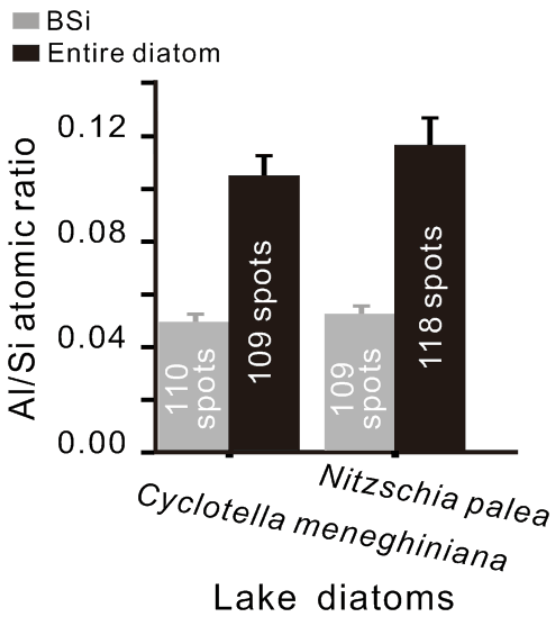

Supplementary Figure 2. Average Al/Si atomic ratios of the entire diatoms (black columns) of the two natural lake diatoms and their BSi (grey columns) obtained by EDS-spot analysis based on more than 100 spots (specified in the brackets) for lake diatoms of *Cyclotella meneghiniana* and *Nitzschia palea* or their corresponding BSi. Error bounds were obtained on the basis of 95% confidence interval for the analysis on Al/Si atomic ratios. Source data are provided as a Source Data file.

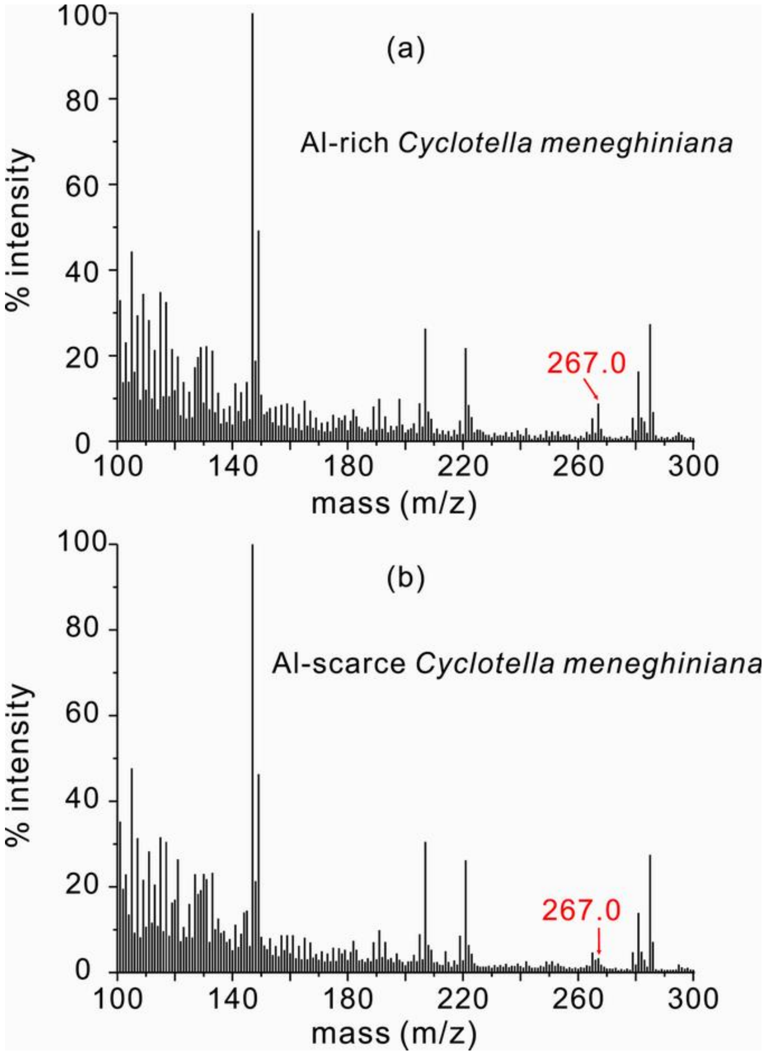

Supplementary Figure 3. Mass spectra of derivative liberated from *Cyclotella meneghiniana*: (a) Al-rich *Cyclotella meneghiniana* and (b) Al-scarce *Cyclotella meneghiniana*. Source data are provided as a Source Data file.

The mass spectrometry (MS) was used to examine the Al state in the study by a Model 2100 PHI TRIFT-II TOF-SIMS (Physical Electronics, USA). The primary ion beam is Gallium LMIG with primary beam energy of 25 KeV and the generator current of 2.0 μA. The strongest signal nearly ~1457 (m/z) was used as the internal standard for normalization.

The entire *Cyclotella meneghiniana* diatoms were used for MS analysis, and two specimens of the cultured diatoms were characterized. One is the Al-rich diatom (denoted Al-rich *Cyclotella meneghiniana*), which was collected from the culture system where the Al concentration is 2.0 μM. This specimen possesses high Al content

(with average Al/Si atomic ratio of 0.072) in the entire diatoms. The other is the Al-scarce diatom (Al-scarce *Cyclotella meneghiniana*), with low Al/Si atomic ratio (< 0.001). It was obtained after successive culturing and harvesting of the initial *Cyclotella meneghiniana* specimen over many generations.

The signal of mass (m/z) = 267 in the carbon MS spectra (Supplementary Figure 3) shows the remarkable difference between Al-rich *Cyclotella meneghiniana* and Al-scarce *Cyclotella meneghiniana*. Such signal in the spectrum of Al-rich *Cyclotella meneghiniana* is distinct (Supplementary Figure 3a), but it almost disappears in the spectrum of Al-scarce *Cyclotella meneghiniana* (Supplementary Figure 3b). Therefore, this difference is attributed to the difference of Al content. In another word, this signal corresponds to the organic components of diatoms that are associated with Al. Based on the carbon species database attached with the Model 2100 instrument, this mass exactly matches the m/z value for $C_8H_{12}N_4O_3SiAl$.

In term of previous studies [1-6], it is possible the transportation of Al for fusion into biogenic silica of diatoms may be accompanied with the formation of frustules using the polycationic polypeptides called silaffins or long-chain polyamines (LCPAs), the critical components of the silica deposition vesicle (SDV). However, identifying the exactly preferential binding sites of Al ions in the organic components is difficult and beyond the scope of this work.

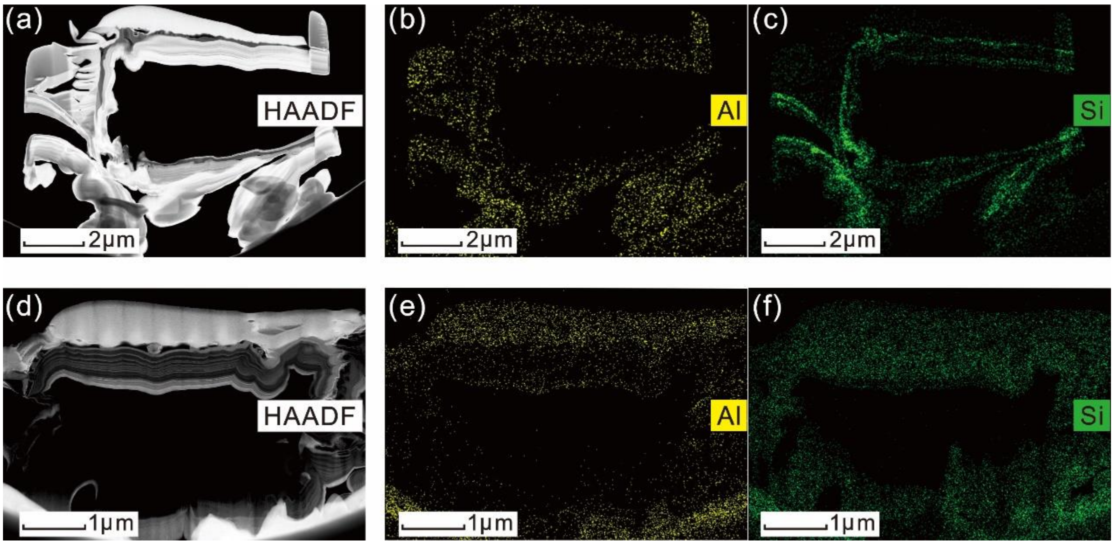

Supplementary Figure 4. The high-angle annular dark field (HAADF) images and the Si and Al distribution results determined by energy-dispersive X-ray spectroscopy (EDS). (a) HAADF images of the sliced fragment of a frustule of BSi-*C*; (b) elemental distributions of Al (yellow shading) for BSi-*C* and (e) for BSi-*N*, respectively; (c) elemental distributions of Si (green shading) for BSi-*C* and (f) for BSi-*N*, respectively; (d) HAADF images of the sliced fragment of a frustule of BSi-*N*. The abovementioned characterizations were performed on sliced fragments of the frustules from the lake sediments using focused ion beam (FIB, FEI Helios NanoLab 450S with a FlipStage equipped with *in situ* scanning transmission electron microscope (STEM) detector). FIB-STEM-EDS mapping analysis instead of EDS spot analysis was used for characterizing the Al and Si distribution in the internal structure of frustules and the average Al/Si atomic ratios. The objective of such characterization is to avoid the disturbance of the abundant impurities existing on the surface and in the pores of frustules in the lake sediments, due to that common chemical pretreatment used for living diatoms does not effectively work for the removal of these impurities from sedimentary frustules. The frustule for characterization was picked up using a nanomanipulator (Oxford OmniProbe 200) from lake sediments and a 5 kV focused gallium ion (Ga^{+}) beam with a beam current of 40 pA was used. The thickness of the fragment is ~80 nm. The Al and Si distributions were obtained using energy-dispersive X-ray imaging attached to a transmission electron microscope (FEI Talos F200 TEM/EDS microscope) with a voltage of 200 kV and a current of 1 nA.

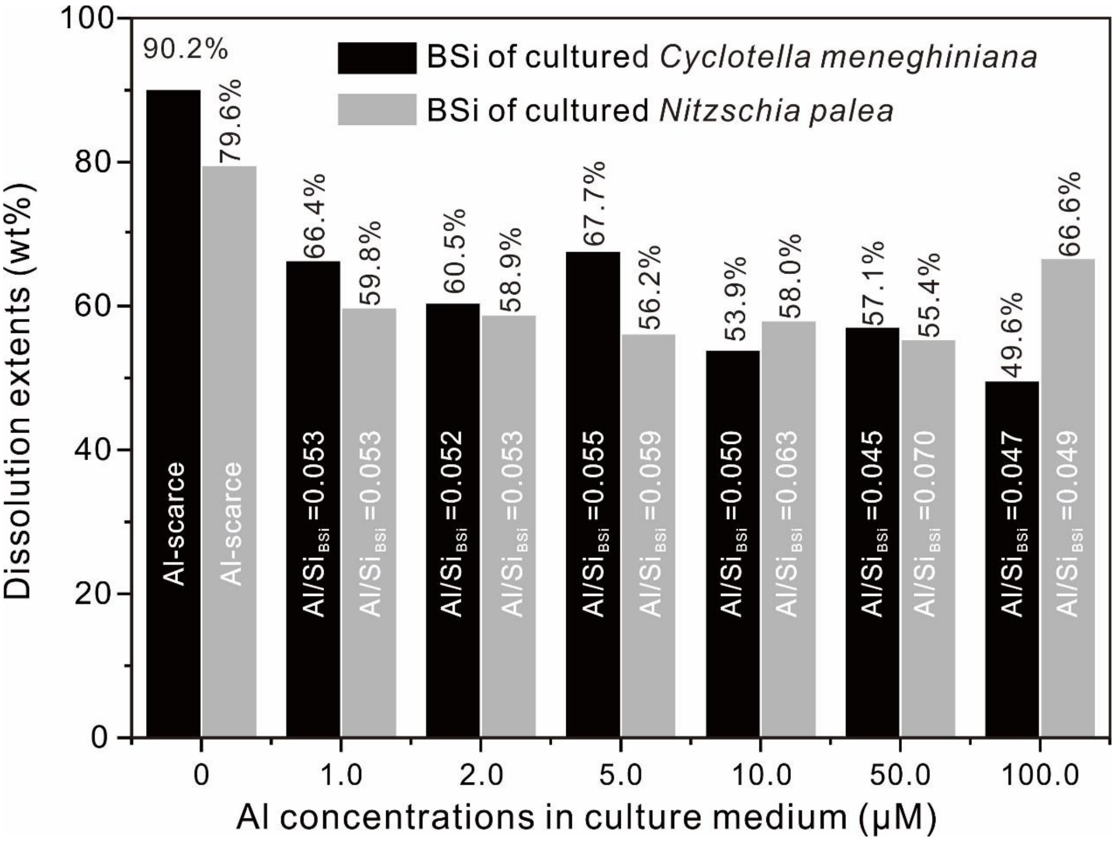

Supplementary Figure 5. The 10-day dissolution extents (wt%) of the BSi extracted from cultured *Cyclotella meneghiniana* and *Nitzschia palea* with various Al/Si atomic ratios. Source data are provided as a Source Data file.

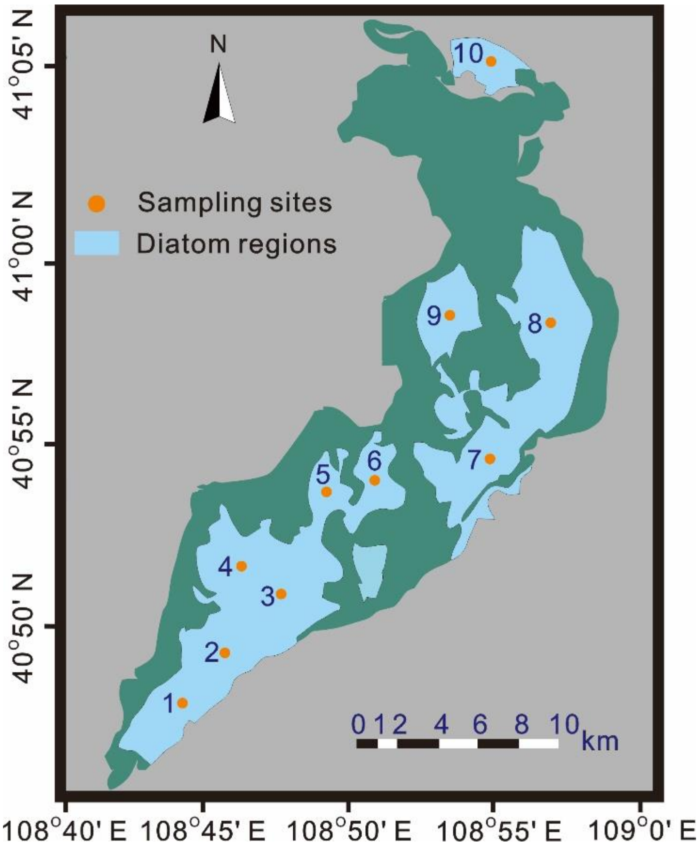

Supplementary Figure 6. Location of sampling sites within Wuliangsuhai Lake.

Wuliangsuhai Lake is the largest freshwater lake in the valley of the Yellow River and also the eighth largest freshwater lake of China, located in Wulate County, Inner Mongolia, China (108°42'–108°58' E, 40°46'–41°7' N). The catchment of Wuliangsuhai Lake is approximately 333.48 km^2, and its water volume ranges from ~2.5×10^8 to ~3.0×10^8 m^3. Precipitation and farmland drainage are main inputs of water to the lake.

Planktons in Wuliangsuhai Lake are dominated by diatoms. More than twenty genera of diatoms are found in the lake, of which *Cyclotella meneghiniana* is dominant, and mainly distributed in areas (approximately 110 km^2) rich in submerged plants. Diatom samples were collected from 10 sampling sites (Supplementary Figure 6).

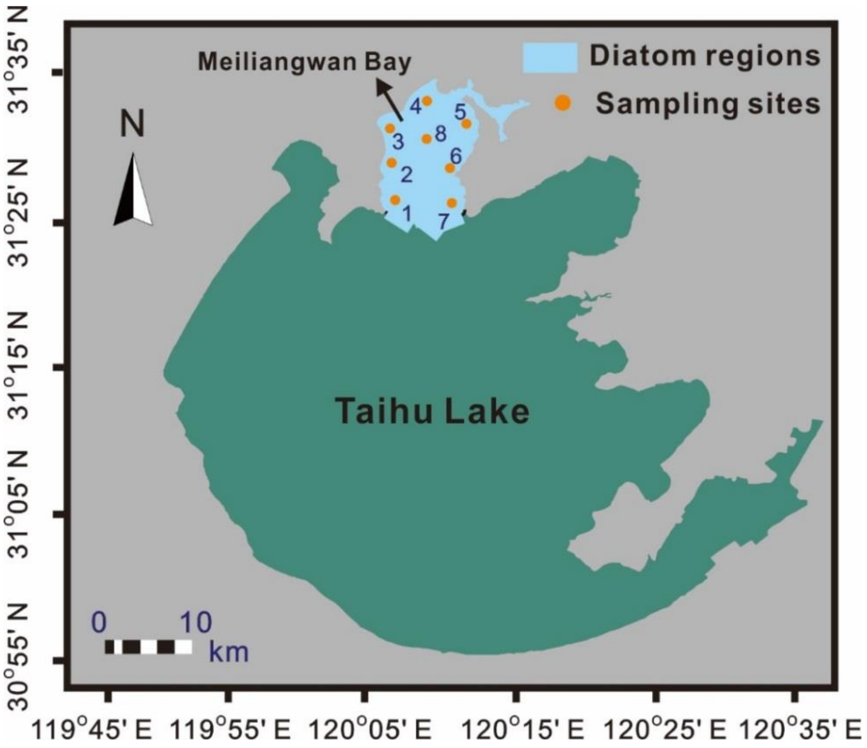

Supplementary Figure 7. Location of sampling sites within Taihu Lake.

Taihu Lake (30°55'–31°32' N, 119°52'–120°36' E) is China's third largest freshwater lake, located on the border between Jiangsu and Zhejiang provinces. The lake catchment is approximately 2338 km^2, and its water volume is 4.43 $\times 10^{10}$ m^3. The sampling sites are mainly located in Meiliangwan Bay, which lies at the northern end of the Lake. The lake water area of this bay exceeds 100 km^2. The *Nitzschia palea* samples were collected from 8 sampling sites of Taihu Lake (Supplementary Figure 7).

Sediment samples were collected at the sampling sites of lake diatoms. Undisturbed sediment cores were obtained using a Beeker sampler (Eijkelkamp Company, Netherland), which has thin-walled tubes with a diameter of 5.7 cm and a length of 150 cm. The sampler tube was inserted into the sedimentary layer and the obtained core was retained in the tube by an inflatable valve in the sampler. The sediment cores were sectioned into 2-cm slices. Then, the profile sections with depths > 2 cm were used and the Al/Si atomic ratios of BSi in the sediments were determined by the FIB-EDS analysis.

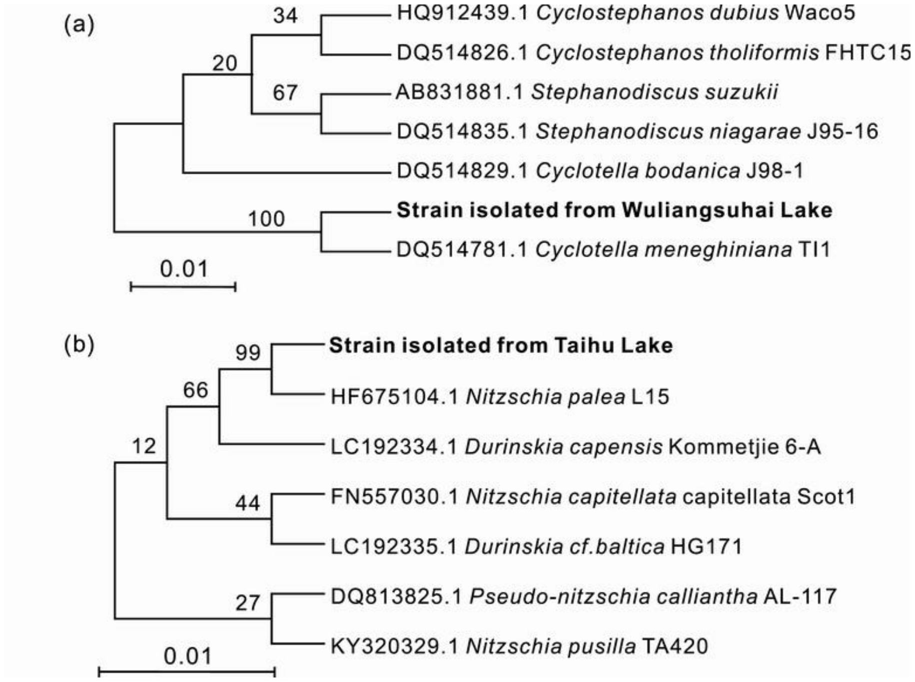

Supplementary Figure 8. Phylogenetic trees of diatom strains isolated from (a) Wuliangsuhai Lake and (b) Taihu Lake. The species of the natural lake diatom samples were identified by determining their rbcL gene sequences [7]. The scale bar indicates the number of substitutions per site for a unit branch length.

The species of the natural lake diatom samples were identified by determining their rbcL gene sequences [7]. The obtained phylogram clearly showed that the diatoms collected from Wuliangsuhai Lake and Taihu Lake are diatom *Cyclotella meneghiniana* (Supplementary Figure 8a) and diatom *Nitzschia palea* (Supplementary Figure 8b), respectively.

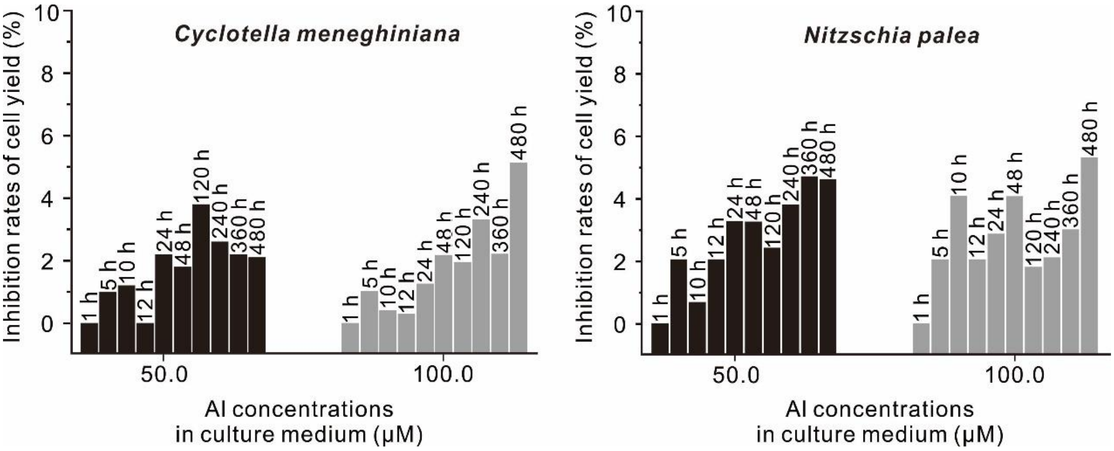

Supplementary Figure 9. Inhibition rates of diatoms cell yield in culturing experiments with initial Al concentrations of 50.0 and 100.0 μM and exposure time (1, 5, 10, 12, 24, 48, 120, 240, 360 and 480 h, respectively). The growth inhibition rates of both diatoms are lower than 5.4%, indicating the high tolerance against Al of the two lake diatoms. Source data are provided as a Source Data file.

The potential growth inhibition of the two freshwater diatoms due to the presence of Al with high Al concentrations of 50.0 and 100.0 μM was evaluated. The growth inhibition rates were calculated based on the diatom cell concentrations in medium with and without Al at different exposure time during the 20 day culturing [8].

$$\text{Inhibition rate}\,(\%) = \frac{(C - C_{\text{Al}})}{C} \times 100$$

Where, C (cell mL^{-1}) is the cell concentration of diatoms which were cultured in Al-free mediums at different culture time;

C_{Al} (cell mL^{-1}) is the cell concentration of diatoms which were cultured in Al-added mediums at different culture time.

Cell counting was performed by flow cytometry (BioMarine-IA 1000, Countstar Company, Shanghai, China). Cultivation experiments followed the processes described in the Methods section and all the parameters of diatom cultivation were in accordance with those of previous experiments.

Supplementary Tables

Sample	Linear fitting formula based on the Si and Al atomic percentages	Correlation coefficients
Cyclotella meneghiniana	y=0.0653x+0.0527	R=0.70<br>R^2=0.48
Nitzschia palea	y=00817x+0.0448	R=0.84<br>R^2=0.70
BSi-*C*	y=0.0346x+0.0254	R=0.91<br>R^2=0.84
BSi-*N*	y=0.0515x-0.0002	R=0.86<br>R^2=0.75

Supplementary Table 1. Linear fitting formulas relating the Si and Al atomic percentages in BSi and the corresponding diatoms.

Sampling location	Diatom species collected in lakes	pH	Average dissolved Al concentration (μM)
Wuliangsuhai Lake	*Cyclotella meneghiniana*	8.5	1.1
Meiliangwan Bay (Taihu Lake)	*Nitzschia palea*	8.2	2.1

Supplementary Table 2. Average pH values and dissolved Al concentrations of the lake water at the diatom sampling locations.

Components	Concentrations
$Ca(NO_3)_2 \cdot 4H_2O$	150 µg L^{-1}
KNO_3	100 µg L^{-1}
$MgSO_4 \cdot 7H_2O$	40 µg L^{-1}
Disodium *β*-ycerophosphate$\cdot 5H_2O$	25 µg L^{-1}
Vitamin B12	0.1 ug L^{-1}
Biotin	0.1 ug L^{-1}
Thiamine HCl	10 ug L^{-1}
HEPES (4-hydroxyethyl piperazine sulfonic acid)	0.5 g L^{-1}
$Na_2SiO_3 \cdot 9H_2O$	0.1 g L^{-1}
Na_2EDTA	0.75 g L^{-1} dH_2O
$MnCl_2 \cdot 4H_2O$	0.041 g L^{-1} dH_2O
$ZnCl_2 \cdot 7H_2O$	0.005 g L^{-1} dH_2O
$Na_2MoO_4 \cdot 2H_2O$	0.004 g L^{-1} dH_2O
$FeCl_3 \cdot 6\ H_2O$	0.097 g L^{-1} dH_2O
$CoCl_2.6\ H_2O$	0.002 g L^{-1} dH_2O

Supplementary Table 3. Components and their concentrations in the diatom medium

Supplementary References

1. Kröger, N., Deutzmann, R. & Sumper, M. Polycationic peptides from diatom biosilica that direct silica nanosphere formation. *Science* **286**, 1129 (1999).
2. Kröger, N., Deutzmann, R., Bergsdorf, C. & Sumper, M. Species-specific polyamines from diatoms control silica morphology. *Proc. Natl Acad. Sci.* **97**, 14133 (2000).
3. Kröger, N., Lorenz, S., Brunner, E. & Sumper, M. Self-assembly of highly phosphorylated silaffins and their function in biosilica morphogenesis. *Science* **298**, 584 (2002).
4. Kröger, N. & Poulsen, N. Diatoms-from cell wall biogenesis to nanotechnology. *Annual Review of Genetics* **42**, 83 (2008).
5. Sumper, M. & Lehmann, G. Silica pattern formation in diatoms: Species−specific polyamine biosynthesis. *ChemBioChem* **7**, 1419-1427 (2006).
6. Wetherbee, R. The diatom glasshouse. *Science* **298**, 547-547 (2002).
7. E. Couradeau, K. Benzerara, E. Gerard, D. Moreira, S. Bernard, G. E. Brown, P. Lopez-Garcia, An early-branching microbialite cyanobacterium forms intracellular carbonates. *Science* **336**, 459-462 (2012).
8. Xie, J. *et al.* Analysis of the proteome of the marine diatom *Phaeodactylum tricornutum* exposed to aluminum providing insights into aluminum toxicity mechanisms. *Environ. Sci. Technol.* **49**, 11182-11190 (2015).
